# Root cause prediction for failures in semiconductor industry, a genetic algorithm–machine learning approach

**DOI:** 10.1038/s41598-023-30769-8

**Published:** 2023-03-27

**Authors:** Abbas Rammal, Kenneth Ezukwoke, Anis Hoayek, Mireille Batton-Hubert

**Affiliations:** Ecole des Mines de Saint-Etienne, Mathematics and Industrial Engineering, Organisation and Environmental Engineering, Henri FAYOL Institute, 42023 Saint-Etienne, France

**Keywords:** Information technology, Scientific data

## Abstract

Failure analysis has become an important part of guaranteeing good quality in the electronic component manufacturing process. The conclusions of a failure analysis can be used to identify a component’s flaws and to better understand the mechanisms and causes of failure, allowing for the implementation of remedial steps to improve the product’s quality and reliability. A failure reporting, analysis, and corrective action system is a method for organizations to report, classify, and evaluate failures, as well as plan corrective actions. These text feature datasets must first be preprocessed by Natural Language Processing techniques and converted to numeric by vectorization methods before starting the process of information extraction and building predictive models to predict failure conclusions of a given failure description. However, not all-textual information is useful for building predictive models suitable for failure analysis. Feature selection has been approached by several variable selection methods. Some of them have not been adapted for use in large data sets or are difficult to tune and others are not applicable to textual data. This article aims to develop a predictive model able to predict the failure conclusions using the discriminating features of the failure descriptions. For this, we propose to combine a Genetic Algorithm with supervised learning methods for an optimal prediction of the conclusions of failure in terms of the discriminant features of failure descriptions. Since we have an unbalanced dataset, we propose to apply an F1 score as a fitness function of supervised classification methods such as Decision Tree Classifier and Support Vector Machine. The suggested algorithms are called GA-DT and GA-SVM. Experiments on failure analysis textual datasets demonstrate the effectiveness of the proposed GA-DT method in creating a better predictive model of failure conclusion compared to using the information of the entire textual features or limited features selected by a genetic algorithm based on a SVM. Quantitative performances such as BLEU score and cosine similarity are used to compare the prediction performance of the different approaches.

## Introduction

The development of microelectronic technologies provides new opportunities to improve the maintenance of production equipment from both the technical and managerial perspective. To establish this improvement in production, it is necessary to focus on an important step that is the failure analysis. This process is a technical procedure for studying how materials and products fail. It is important to understand how and why a component fails when it no longer performs its intended function^1^. The main goal of failure analysis is to find the underlying root cause of the failure, ideally with a view to removing it and identifying ways to prevent it from happening again. Objective failure analysis can have a number of good outcomes such as obtaining an information database that can be put to good use in preventing future failures, enhancing the quality and extending the life of products and services, and making the most of the economic aspects^2^. To meet these principal fundamental challenges in our digital world, it is important to build an information database to describe failures and their conclusions, allowing to ensure that increasingly complex electronic systems operate reliably and securely.

Many organizations use the Failure Reporting, Analysis, and Corrective Action System (FRACAS) to keep track of product problems. The FRACAS technique’s main tasks are^3^: recording and capturing information about failures and problems, provide new information to support future reliability analyses, provide report summaries of incident counts and provide failure dataset and metrics to measure quality parameters. Developing a novel technique based on artificial intelligence (AI) to swiftly assess and discover faults during the development and manufacture of electronic components and systems, using the final report generated by FRACAS, is one of the key difficulties facing our digital world. The incorporation of AI and multi-structured data sources is critical to the success of data-driven maintenance. When an AI-enhanced technique is introduced and integrated into a reliability-centered maintenance analysis of complex production systems, failure rates are reduced, and availability is improved^4^.

Text mining is an artificial intelligence (AI) technique that applies natural language processing (NLP) to convert unstructured text in documents and databases into normalized, structured data that can be analyzed or used to train machine learning (ML) algorithms^5^. Text mining is also a technique for extracting information from unstructured documents and identifying novel and previously unknown patterns. Then, the next step is feature or attribute selection. This step focuses on deleting elements that are not significant to the mining process^6^. In addition, this step has several advantages: reduce computational complexity; get fewer noises in the decision space and reduce the dimension to have more consistent and homogeneous dataset^7^.

In our study, we have a textual dataset that consists of the description of the failure analysis and the failure conclusion for microelectronic technologies’ products. Our objective is to construct a model able to predict the failure conclusion features from the failure analysis description features. However, not all textual information’s are valuable for the construction of predictive model, while the use of limited number of a priori features may be tricky. Feature selection reduces dimensionality by selecting a subset of original input textual variables. In other words, the textual variable selection strategy decreases the dimension of textual features that may be relevant to a specific phenomenon by identifying the best minimum subset without transforming the data into a new set^8^. In order to achieve complicated models for prediction and classification algorithms, we implement the selection of relevant textual variables while excluding non-informative variables.

Various mathematical techniques have been used to select optimal subsets of variables: successive projections algorithm^9^, backward/forward selection algorithm^10^, reweighted adaptive competitive sampling, importance of variables for projection, elimination of non-informative variables^11^, interval partial least squares regression^12^, Monte Carlo-elimination of non-informative variables^13^, Particle Swarm Optimization and Deep Learning Approach^14^, feature learning enhanced convolutional neural network (FLE-CNN)^15^, competitive adaptive reweighted sampling partial least squares^12^, etc. However, most of these techniques are not well suited to textual datasets. On the other hand, the application of these methods leads to the loss of a lot of information during the analysis.

The genetic algorithm (GA) belongs to research techniques that emulate the principle of natural selection. GA performs a search in complex, large and multimode landscapes, and provides near-optimal solutions for objective or fitness function of an optimization problem^16^. However, the cost of computational time is high because its long string representation evolves in high dimensional space typical for textual data. A genetic algorithm is a bottom-up strategy that chooses the best features subset based on the “survival of the fittest” principle, with each chromosome competing with the others^16^. That is, the quality of chromosomes is assessed using a predetermined fitness function. The fitness function is arguably the most important part of a GA having the role to measure the quality of the chromosome in the population according to the given optimization objective. Supervised learning methods can be used to derive new fitness functions that can transform a textual data in a much lower-dimensional subspace more adequately regarding a specific application^17^. Different types of supervised methods exist in the literature. The best-known are Decision Tree model (DT), and Support Vector Machine model (SVM). A study was conducted to demonstrate that the combination of genetic algorithm and support vector machine method improves the textual classification accuracy of the spam dataset^18^. Another study shows that the efficiency of feature selection based on information gain and genetic algorithm can reduce the dimension of the text vector and improve the accuracy of text classification^19^. A recent paper proposes the genetic algorithm-oriented latent semantic feature methodology to achieve better representation of documents in text classification^20^.

Therefore based on all the above, one can summarize the motivation of combining GA and supervised learning methods by the following:

The combination of Genetic Algorithms (GA) and supervised learning methods has been a popular topic of research in the field of machine learning and optimization. For example, in a study by Fernández et al. (2002), the authors used a GA to optimize the parameters of a support vector machine (SVM) for a classification task and showed that the combination of these two approaches led to improved performance compared to using only the SVM. Another study by Liu et al. (2011) proposed a GA-based approach for feature selection in conjunction with a decision tree classifier, showing that the combination of these two methods outperformed individual methods in several benchmark datasets. In addition to parameter optimization, GAs have also been used to search for optimal network architecture in deep learning. For instance, Real et al. (2017) proposed a method called “Large-Scale Evolution of Image Classifiers” where they used a GA to evolve the architecture of Convolutional Neural Networks (CNNs) and showed that the evolved architectures outperformed manually designed ones in the CIFAR-10 and CIFAR-100 image classification benchmarks. These studies demonstrate the potential of combining GA and supervised learning methods for improving performance in various applications, and highlight the need for further research in this area.

On the other hand, the research gaps and challenges and how we are overcoming these points can be summarized by the following:

The most challenging issues in this study are likely related to the task of developing a predictive model that can accurately predict the failure conclusions based on the failure descriptions. This is a challenging task as it requires the model to learn the relationship between the input features and the target output, which can be difficult due to the presence of noisy or irrelevant features, imbalanced class distributions, and non-linear relationships between features and target.

The proposed method addresses these challenges by combining a genetic algorithm with a decision tree classifier, referred to as GA-DT. The GA is used to search for a subset of the most discriminative features from the failure descriptions, which are then used as input to the decision tree classifier. By doing so, the GA helps to overcome the issue of noisy or irrelevant features, as it only selects the most informative features for the classifier to use. Additionally, decision trees are known to be able to handle imbalanced class distributions and non-linear relationships, making them a suitable choice for this task.

The effectiveness of the proposed GA-DT model is demonstrated through experiments, which show improved performance compared to using only a decision tree classifier or only a genetic algorithm. This highlights the contribution of the proposed method, which combines the strengths of both GA and decision tree classifiers to improve the accuracy of the predictive model.

Then, the main goal of this study is to build an advanced predictive model capable of predicting failure outcomes significantly using failure analysis description. Another objective is to investigate the potential of using a supervised variable selection technique using a genetic algorithm to identify more informative and useful text features from the text dataset that contains a very large number of words, and to show whether the features selected by the proposed method can significantly improve the performance of predictive models between failure conclusion features and failure analysis description features. We propose a methodology based on the association of a genetic algorithm with a supervised model such as the decision tree or support vector machine evaluated by the F1 score as a fitness function for the identification of the discriminating variables applied the failure analysis textual data. This function allows calculating the accuracy of predictive models applied on unbalanced dataset. The suggested algorithms are called GA-SVM and GA-DT.

This article is structured as follows: In the second part, we present what Feature Selection is and its associated algorithms. Then, we detail the operating principle of population-based metaheuristic algorithms. We focus more particularly on Genetic Algorithm, and their detailed operation that allows the selection of relevant features. In this part of this work, we present machine-learning algorithms used to calculate the fitness value for the metaheuristic algorithms. We go deep in the description of supervised methods such as Support Vector Machine (SVM), and Decision Tree (DT). In the third part, we present the results obtained by applying our metaheuristic-machine learning algorithms combination on the failure conclusion features and failure analysis description features. We show that the results observed allow us to pick the most valid model, which is the GA-DT, confirmed with the different metrics as BLEU score and cosine similarity at a division of $70\%$ training set and $30\%$ testing set. Finally, and after discussing the results, we close with a general conclusion on the interest of the combination of feature selection algorithms with machine learning methods, its capability and performance in dimension reduction, and on the possibilities of implementing other tools belonging to metaheuristic algorithms to improve the accuracy rates.

## Mathematical methods

### Supervised learning modelling

#### Multiclass error-correcting output codes (ECOC) model using support vector machine (SVM)

The Error-Correcting Output Codes (ECOC) framework is a basic yet effective method for dealing with the multi-class categorization problem based on the embedding of binary classifiers, where the classifier consists of multiple binary learners such as support vector machines (SVMs). The ECOC model classifiers allow to store training data, parameter values, prior probabilities, and coding matrices^21^. These classifiers aims to perform tasks such as predicting labels or posterior probabilities for new data. The multi-class ECOC model using SVM methods consist of three major components that are encoding, binary classifier learning, and decoding steps. In the coding procedure, a coding matrix is usually first determined for several classes, where each row of the coding matrix represents a specific class. Then, a group of independent binary classifiers is formed based on a different partition of the original data according to each column of the coding matrix. Finally, a new data is predicted as a specific class through the decoding procedure based on the outputs of the learned binary classifiers and the coding matrix.

Let $X=\{x_{j} \}_{j=1}^{n}$ be a training set of *n* samples of observed variables, where a d-dimensional vector represents each sample, and let *C* be an unobserved random variable denoting the class membership of $x_{j}$, where $C \in \{c_{1}, \ldots , c_{k}, \ldots , c_{K} \}$ with *K* denoting the number of class. In $k^{th}$ class SVM problem, class $c_{k}$ is separated from the remaining classes. All *k* binary SVM classifiers are combined together to make a final multi-class classifier. Here the remaining means that all the data points from classes other than $c_{k}$ are combined to form one class $c_{l}$. The optimal hyperplane that separates data points from the class $c_{k}$ and the combined class $c_{l}$ is found by using the standard SVM approach. We denote the optimal separating hyperplane discriminating the class $c_{i}$ and the combined class $c_{k}$ as^22^:

1 $$\begin{aligned} g_{k}(x_{j}) = w_{k} \times \phi (x_{j}) + b_{k} \quad k \in \{1, \ldots , K \}. \end{aligned}$$

where $w_{k} \in {\mathbb {R}}^{S}$ is the weight vector, *b* is the bias and the mapping function $\phi$ projects the training data into a suitable feature space ${\mathbb {R}}^{S}$ to allow for nonlinear decision surfaces. The parameters of the decision function $g_{k} (x_{j})$ are determined by the following minimization^23^:

2 $$\begin{aligned} \min J(w_{k}, \xi ) = \frac{1}{2}\Vert w_{k} \Vert ^{2} + C \sum _{j=1}^{n} \xi _{j}. \end{aligned}$$

subject to

3 $$\begin{aligned} y_{j}\left( w_{k}^{T}\phi (x_{j}) + b_{k}\right) \ge 1 - \xi _{j} \quad \xi _{j} \ge 0; j = 1 \ldots n. \end{aligned}$$

with scalar $y_{j} \in \{-1,+1\}$ denoting its class label, $C \in {\mathbb {R}}^{+}$ is a regularization constant and $\xi _{j}$ denote a slack variable can be introduced to relax the separability constraints in Eq. (2).

The decision rule $f_{k} (x_{j})$ that assigns the vector $x_{j}$ to the class $c_{k}$ given by:

4 $$\begin{aligned} f_{k} (x_{j}) = \text {sign}(g_{k} (x_{j})). \end{aligned}$$

The main difficulty in this approach is that the outputs of the classifiers $f_{k} (x_{j})$ are binary values. The usual way to handle this problem is to ignore the sign-operator in Eq. (4). After finding all the optimal hyperplanes given by $g_{k} (x_{j})$ for $k \in \{1, \ldots , K \}$, we say $x_{j}$ is in the class which has the largest value of the decision function and is given by^24^:

5 $$\begin{aligned} \hat{c_{k}}(x_{j}) = \underset{k}{{\text {argmax}}} \left( g_{k} (x_{j})\right) . \end{aligned}$$

In this approach the index of the largest component of the discriminant functions $g_{k} (x_{j})$ for $k \in \{1, \ldots , K \}$ is assigned to the data point $x_{j}$. The error rate ${\mathcal {R}}^{SVM}$ of the SVM classifier, which is defined as:

6 $$\begin{aligned} {\mathcal {R}}^{SVM}(x_{j}) = \frac{1}{n} \sum _{j=1}^{n} 1_{c_{k} \ne \hat{c_{k}}}. \end{aligned}$$

with $x_{j}$ that belongs to the class $c_{k}$ estimated by the method classifier in the class $\hat{c_{k}}$ and $1_{c_{k} \ne \hat{c_{k}}} (x_{j})$ is the indicator function defined as:

7 $$\begin{aligned} 1_{c_{k} \ne \hat{c_{k}}} (x_{j}) = {\left\{ \begin{array}{ll} 1, &{} \text{ if } \text{ class } c_{k} \ne \hat{c_{k}} \\ 0, &{} \text{ if } \text{ class } c_{k} = \hat{c_{k}} \end{array}\right. } \end{aligned}$$

#### Decision tree classifier

A decision tree classifier is a non-parametric classifier that does not require any a priori statistical assumptions to be made regarding the underlying distribution of data. The basic structure of the decision tree, however, consists of one root node, a number of internal nodes and finally a set of terminal nodes. A node is a subset of the predictors that is used to determine a split. A non-terminal node or parent node is a node that is further split into two child nodes. Growing a tree consists of selecting the optimal splits to determine a non-terminal node, and the assignment of each terminal node to a class^25^. The data is recursively divided down the decision tree according to the defined classification framework.

Classes are simply assigned to a terminal node by observing which class is mostly commonly observed in that region of the tree. Thus, the challenge is to optimally choose the best variable and split that variable to maximize the purity or similarity among the responses. The impurity of a parent node $\tau$, denoted $i(\tau )$, is zero when all observations are in the same class. A split *s* is determined by selecting the best predictor and split value that optimizes the highest reduction in purity^26^:

8 $$\begin{aligned} \Delta (s, \tau ) = i(\tau ) - \sum _{b=1}^{B} p(\tau _{b}/ \tau ) i(\tau _{b}). \end{aligned}$$

where $\tau _{b}$ denotes child node *b*, $p(\tau _{b}/ \tau )$ is the proportion of observations in $\tau$ that are assigned to $\tau _{b}$, and *B* is the number of branches after splitting. Two common impurity functions are the entropy criterion^26^:

9 $$\begin{aligned} i(\tau ) = - \sum _{k=1}^{K} p_{k} \log _{2} (p_{k}). \end{aligned}$$

and the Gini index criterion

10 $$\begin{aligned} i(\tau ) = - \sum _{k=1}^{K} p_{k}^{2}. \end{aligned}$$

where $$ p_{k}$$ is the proportion of observations in class $$c_{k}$$ with $$ k \in \{1, \ldots , K \}$$. Pruning is based upon successive steps of removing lower branches that lead to improved classification rates. Once the final tree is determined by $$\Delta (s, \tau )$$, it is natural to evaluate its predictive performance by comparing the observed class to the predicted class for observation $$x_{j}$$. In a terminal node *m*, representing a region $$R_{m}$$ with $$n_{m}$$ observations, let

11 $$\begin{aligned} \hat{p_{mk}} = \frac{1}{n_{m}}\sum _{j=1}^{n_{m}} 1_{C_{k}}(x_{j}). \end{aligned}$$

denote the proportion of class $c_{k}$ observations in terminal node *m*^27^. We classify the observations in node *m* to class

12 $$\begin{aligned} \hat{c_{k}}(x_{j}) = \underset{k}{{\text {argmax}}} (\hat{p_{mk}}). \end{aligned}$$

The misclassification error rate is simply the proportion of observations in the node that are not members of the majority class in that node.

13 $$\begin{aligned} {\mathcal {R}}^{DTC}(x_{j}) = \frac{1}{n} \sum _{j=1}^{n} \left( 1- \max _{k}(\hat{p_{mk}}(x_{j}))\right) . \end{aligned}$$

### Supervised learning modelling

**Figure 1:**
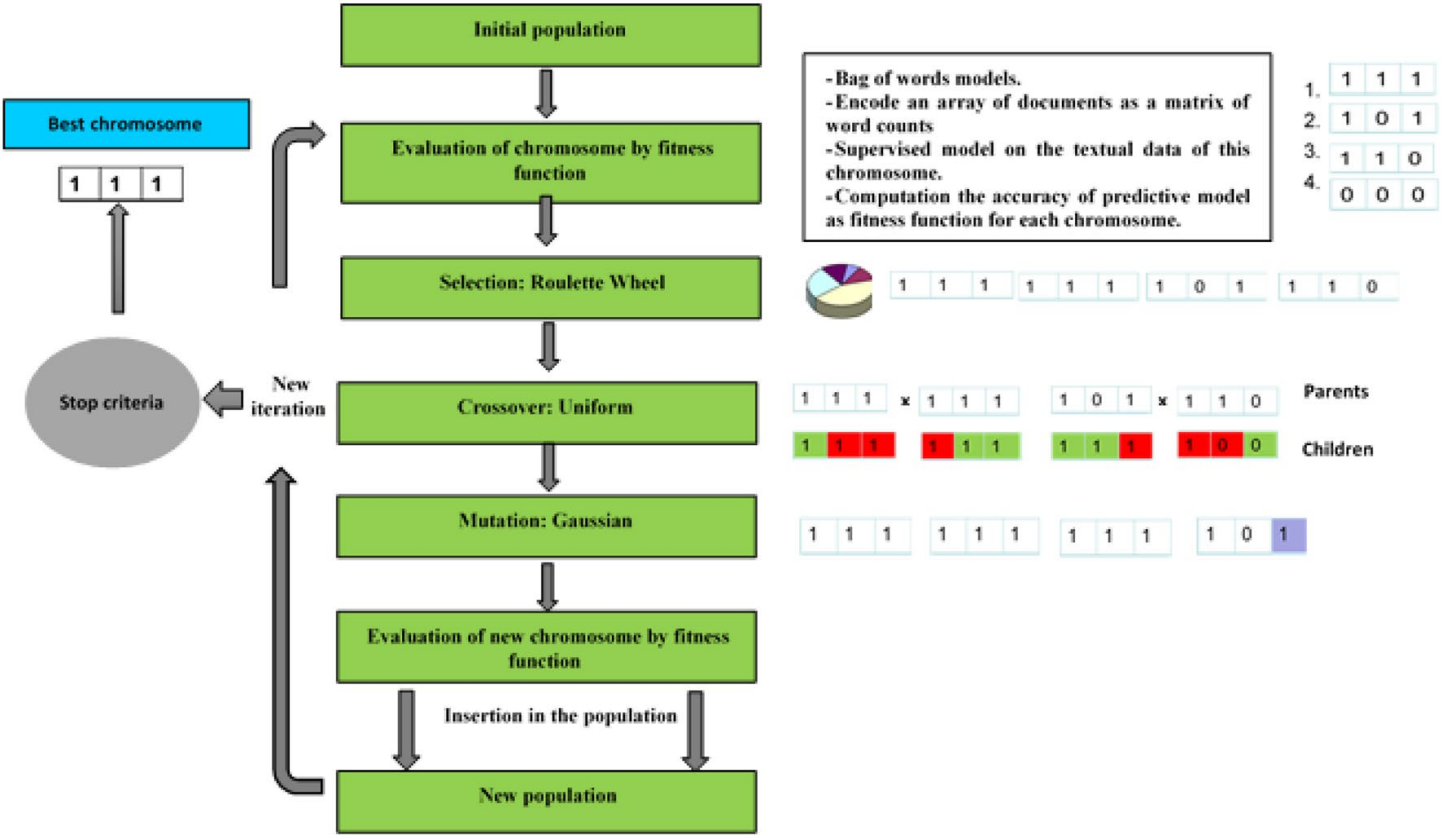
Synoptic representation of the proposed GA methodology.

Genetic algorithms (GA) are a type of evolutionary optimization computation that became popular through the work of Holland^28^. These algorithms are based on the concept of natural selection of solutions by copying its main principles. Each solution may be considered as a population where each element is represented in the form of a chromosome, with selected textual feature positioned as genes^28^. The GA steps reproduce the various evolutionary operations such as crossover and mutation allowing to select for each generation the best chromosomes and to identify at the end an optimal chromosome with respect to an optimization criteria defined by a fitness function^29^. Figure 1 shows the steps of the informative feature selection procedure using a GA^30^.

The GA can be applied on data matrix $X=\{x_{j} (y)\}_{j=1}^{n}$ with $x_{j }(y) \in {\mathbb {R}}^{d}$ and y is the set of textual features for failure description dataset. This procedure gives in each one of these cases an optimal chromosome $z_{0}=[z_{01}\cdots z_{0l} \cdots z_{0L} ] \in {\mathbb {R}}^{L}$ with $z_{0l}$ textual feature form *y* and *L* the number of variables chosen to select. The optimal chromosome allows extracting a new sub-data matrix $\{x_{j} (z_{0}) \}_{j=1}^{n}$ of under-dimensioned data on which we can apply methods of data analysis. The GA steps are briefly described thereafter, being detailed in the articles^31^ and^32^.

1. Initialization: The initial parameters are : the chromosome size *L* (the number of genes corresponding to the number of features to be selected in each case) ; the population size *N* (the number of chromosomes per generation) ; the number of elites $N_{e}$ (the chromosomes with the best fitness values in the current generation that are guaranteed to survive to the next generation) ; the fraction of crossover $F_{c}$ (the number of chromosomes selected to perform crossover $N_{c}$ such that $N_{c} = F_{c} \times (N-N_{e}))$. The stop parameters are: the maximal number of iterations *T* and the tolerance $\epsilon$ for the fitness function. The first step of a GA is the creation of the starting population *P*(0). N chromosomes are generated by randomly selecting L variables from *y* ($L < S$ is the size of the chromosomes):
  14 $$\begin{aligned} P(0) = \left\{ z_{i} = [z_{i1}, \ldots , z_{iq}, \ldots , z_{iL} ] \in {\mathbb {R}}^{L} \right\} _{i=1}^{N}. \end{aligned}$$
  The initial population *P*(0) of wavenumbers variables is chosen randomly from the set of uniformly distributed variables ranging over their maximum and minimum limits^31^ :
  15 $$\begin{aligned} z_{i}^{0} \sim U(z_{i}^{min},z_{i}^{max}). \end{aligned}$$
  where $$z_{i}^{0}$$ signifies the initial $$l^{th}$$ variable of the $$i^{th}$$ population; $$z_{i}^{min}$$ and $$z_{i}^{max}$$ are the minimum and maximum limits of the $$l^{th}$$ decision variable; $$ U(z_{i}^{min},z_{i}^{max})$$ signifies a uniform random variable ranging over$$ [z_{i}^{min},z_{i}^{max}]$$. Then, computation is done over generations. For each generation (*t*), we obtain the population of chromosomes $$\{z_{i(t)} \}_{i=1}^{N}$$ the steps thereafter give another population of chromosomes $$\{z_{i(t+1)} \}_{i=1}^{N}$$.
2. Evaluation: Each chromosome$z_{i(t)}$ is rated by a fitness function *F*(.) that assigns a value $F_{i} = F(z_{i(t)})$. The smaller the $F_{i}$ value is, the more corresponding chromosome will have chance to be selected. The role of a fitness function is to measure the quality of the chromosome in the population according to the given optimization objective^32^. Since we want to create a predictive model between the failure description dataset *X* and the failure conclusion dataset *Y*, we propose to use the supervised model for each chromosome such as the decision tree (DT) and the support vector machine (SVM) and then to calculate the $F_{1}$ score of each model built as a fitness function to assess the qualities of our predictive model obtained. The $F_{1}$ score of these supervised learning methods is one of the simplest methods that can be used as a classical fitness function to evaluate the accuracy of predictive model. The fitness function is defined as follows:
  16 $$\begin{aligned} F_{i}^{\text {model}} = F^{\text {model}}(z_{i}) = \left( 1-F_{1}^{\text {model}}(z_{i}) \right) . \end{aligned}$$
  with
  17 $$\begin{aligned} F_{1}^{\text {model}}(z_{i}) = 2 \times \frac{P_{r}^{\text {model}}(z_{i}) \times R_{c}^{\text {model}}(z_{i})}{P_{r}^{\text {model}}(z_{i}) + R_{c}^{\text {model}}(z_{i})}. \end{aligned}$$
  where $F_{1}^{\text {model}}$ is the$F_{1}$ score defined as the harmonic mean between precision and recall; $P_{r}^{\text {model}}$ is positive predictive value (precision) and $R_{c}^{\text {model}}$ is the sensitivity (Recall) of the predictive model such as SVM and DT. This function ($F_{1}$ score) is very useful when dealing with unbalanced class issues. These are problems when one class can dominate the data set. For each fitness function $F_{i}$, the values are ordered in ascending order and the best $N_{e}$ chromosomes are selected based on this ordering. These surviving chromosomes will be copied unchanged in the next population.
3. Selection: Used for choosing parents from the population for crossing, this step may be implemented in different ways: rank, stochastic, roulette wheel, stochastic universal sampling selection, etc. We have chosen the stochastic universal sampling selection since this method is zero-biased, has no deviation between the expected reproduction rate and the algorithmic sampling frequency, and has a minimum spread^33^. The selection is performed probabilistically so that an individual’s selection probability is proportional to the individual’s fitness. First, we compute the probability $p_{i}$ of selecting the chromosome $z_i$ and the cumulative probability $q_{i}$:
  18 $$\begin{aligned} p_i= & {} \frac{F_i}{\sum _{i=1}^{N}F_i}. \end{aligned}$$
  19 $$\begin{aligned} q_i= & {} \sum _{i=k}^{i}p_k. \end{aligned}$$
  Next, we generate a uniform random number $$r \in [0, \frac{1}{N}]$$. If $$ r < q_1$$ then we select the first chromosome $$z_1$$, otherwise we select the chromosome $$z_i$$ such that $$q_{i-1} < r \le q_i$$. The ascending ordered $$F_{i}$$ values allows selecting $$N_{e}$$ chromosomes guaranteed to survive to the next generation and $$N_{p} = (F_{c} + 1)\times N - 2N_{e}$$ parent chromosomes for the crossover.
4. Crossover: This step attempts to extract genes from the selected chromosomes and recombines them into potentially superior children. We have chosen the uniform crossover since it gives good results in a majority of the cases. A gene is randomly selected either from the first or from the second parent^34^. The crossover operation gives $$N_{c} = (F_{c} \times N) - N_{e}$$ children. To explain the uniform crossover, the parent’s chromosomes $$p_{1} [z_{iq} ]$$, $$ p_{2} [z_{iq} ]$$ and the children chromosomes $$ o_{1} [z_{iq} ]$$, $$ o_{2} [z_{iq} ]$$, $$ q = 1 \ldots L$$ are gene arrays. The most popular crossover variant between real numbers is the uniform crossover. Genes situated in the q position of the children chromosomes $$ z_i$$ are calculated as it follows^35^:
  $\alpha$ is a random vector of real numbers uniformly distributed with the same size as $p_1$, $p_2$, $o_1$, $o_2$ where $\alpha _{q} \in [0,1]$.
  Children are copied from parents and crossover is obtained with Eqs. (20) and (21):
    20 $$\begin{aligned} o_{1} [z_{i} ]= & {} p_{1} [z_{i} ] \quad \text {for each} \quad \alpha _{q} > 0.5, o_{1} [z_{iq} ]=p_{2} [z_{iq} ]. \end{aligned}$$
    21 $$\begin{aligned} o_{2} [z_{i} ]= & {} p_{2} [z_{i} ] \quad \text {for each} \quad \alpha _{q} > 0.5, o_{2} [z_{iq} ]=p_{1} [z_{iq} ]. \end{aligned}$$
5. Mutation: is a genetic operator used the modification of the value of gene to maintain genetic diversity from one generation of a population to the next one. We have chosen the Gaussian operator since it produces the best results for most of the fitness functions^36^. This operator adds a unit Gaussian distributed random value to $N_{p} - 2N_{c}$ chromosomes. The new values of the genes are then rounded to the nearest integer. The standard deviation of this distribution is the parameter that the call “scale” which is equal to one in the first generation, but this parameter is controlled during the next generations by another parameter that is “shrink”. The standard deviation at the tth generation, $\sigma _{t}$ is the same at all coordinates of the parent chromosome, and is given by the recursive formula^37^:
  22 $$\begin{aligned} \sigma _{t} = \sigma _{t-1}\times (1- \text {shrink} \frac{t}{T}). \end{aligned}$$
  Where *T* the number of generations. A low value of “shrink” produce a small decrease in the amplitude of the mutation on the indices of gene positions.
6. Steps 1 to 5 are repeated until the maximal number of iterations *T* is reached or when GA has converged, i.e. the average relative change in the fitness function value is less than the tolerance $\epsilon$. This procedure gives an optimal chromosome $z_{0}$, which depends on the fitness function and the initial values. With the proposed choice of the GA steps, we have found that the same optimal chromosome is found whatever the initial values of chromosomes were used.

### Computing similarities between documents

#### BLEU score

The BiLingual Evaluation Understudy (BLEU) scoring algorithm assesses the similarity between a predictive document and a collection of reference documents. To assess the quality of document translation and summarization models, we use the BLEU score. The n-gram counts, clipped n-gram counts, modified n-gram precision scores, and a brevity penalty are used to calculate the BLEU score^38^.

If necessary, the clipped n-gram counts function Countclip truncates each n-gram gram’s count so that it does not exceed the highest count found in any one reference for that n-gram. The clipped counts function is defined as follows:

23 $$\begin{aligned} \text {Count}_{\text {clip}}(\text {n-gram}) = \min _{n}\left( \text {Count}(\text {n-gram}); \text {maxRef}(\text {n-gram})\right) . \end{aligned}$$

where $\text {Count}(\text {n-gram})$ represent the n-gram counts and $\text {maxRef}(\text {n-gram})$ is the highest n-gram count observed in a single reference document for that n-gram. The updated n-gram precision scores are calculated as follows:

24 $$\begin{aligned} p_{n} = \frac{\sum _{D \in \{ \text {Predictive Document} \}}\sum _{\text {n-gram} \in D} \text {Count}_{\text {clip}}(\text {n-gram}) }{\sum _{D^{'} \in \{ \text {Predictive Document} \}}\sum _{\text {n-gram}^{'} \in D^{'}} \text {Count}_{\text {clip}}(\text {n-gram}^{'})}. \end{aligned}$$

where *n* is the length of n-gram and $\text {Predictive Document}$ is the set of sentences in predictive documents, *D* and $D^{'}$ are predictive documents . Given an n-gram weight vector *w*, the BLEU score formulation is given by^38^:

25 $$\begin{aligned} \text {BLEU score} = \text {BP} \times \exp \left( \sum _{n=1}^{N} w_{n} \log (\bar{p_{n}})\right) . \end{aligned}$$

where *N* is the greatest length of n-grams, $\bar{p_{n}}$ are the geometric means of the modified n-gram precisions, and BP is the brevity penalty defined as

26 $$\begin{aligned} 1_{c_{k} \ne \hat{c_{k}}} (x_{j}) = {\left\{ \begin{array}{ll} 1, &{} \text{ if } \quad c > r \\ \exp (1-\frac{r}{c}), &{} \text{ if } \quad c < r \end{array}\right. } \end{aligned}$$

BLEU score returned as a scalar value in the range [0, 1]. A BLEU score close to zero indicates low similarity between the predictive document and the references. A BLEU score close to one indicates strong similarity. If the predictive document is identical to one of the reference documents, the score is one.

#### Cosine similarity

The similarity of two vectors in an inner product space is measured by cosine similarity. It determines whether two vectors are pointing in the same general direction by measuring the cosine of the angle between them. In text analysis, it is frequently used to determine document similarity^39^. Let us see how documents in our corpus are related to one another. Let $t_{1}$ and $t_{2}$ be two vectors that represent the topic associations of documents $d_{1}$ and $d_{2}$, respectively, where $t_{1}^{(k)}$ and $t_{2}^{(k)}$ are the number of terms in $d_{1}$ and $d_{2}$ that are connected with subject k respectively. The cosine similarity can then be used to calculate a measure of document similarity^39^:

27 $$\begin{aligned} c =\frac{\sum _{k}t_{1}^{(k)} \times t_{2}^{(k)}}{\Vert t_{1} \Vert \times \Vert t_{2} \Vert }. \end{aligned}$$

where $\Vert t_{j} \Vert$ denotes the norm of vector $t_{j}$. Cosine similarity score indicate a scalar value in the range [0, 1]. A cosine similarity close to zero indicates poor similarity between the predictive document and the references. A cosine similarity close to one indicates strong similarity.

### Proposed methodology for evaluation the predictive model of failure analysis

**Figure 2:**
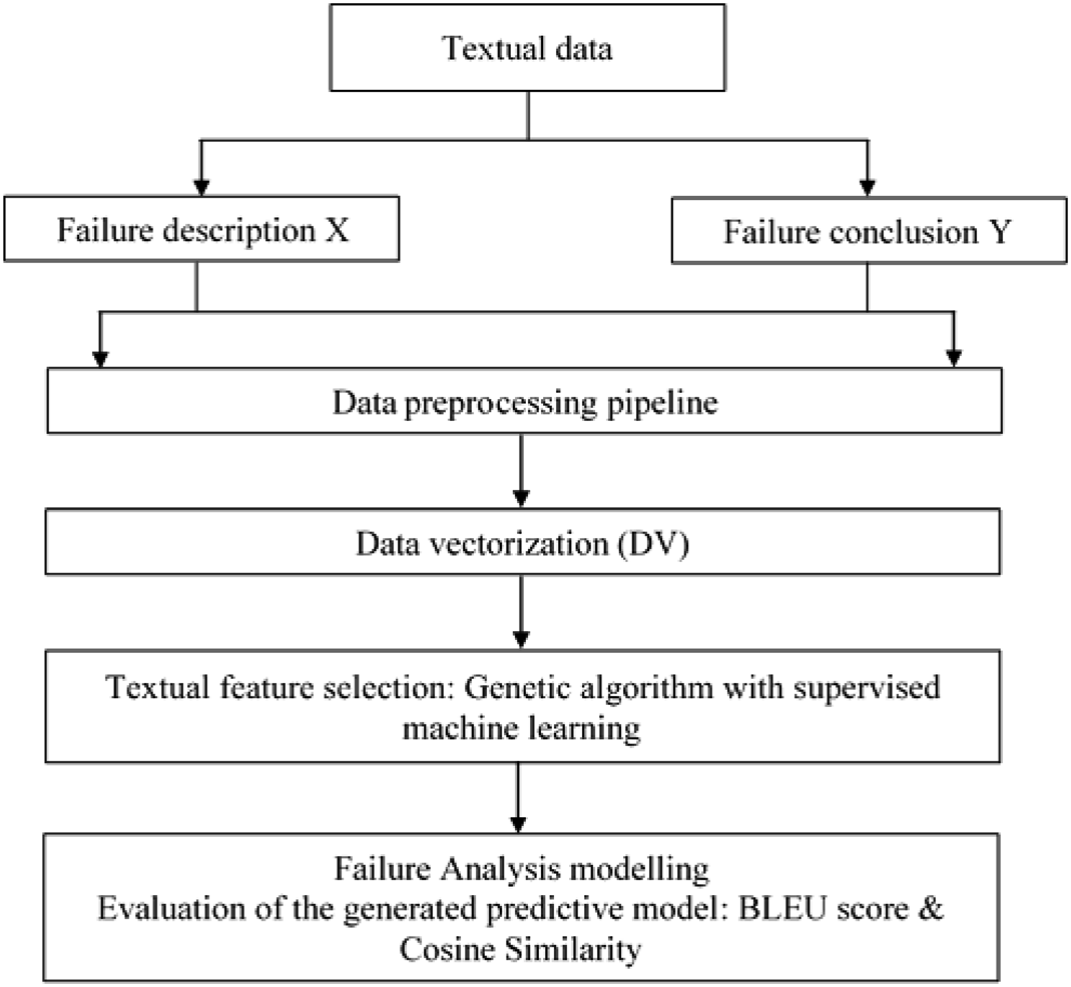
Synoptic representation of the proposed GA methodology.

In this section, we present the methodology proposed for the selection of variables by the genetic algorithm combined with the decision tree (GA-DT) or the support vector machine (GA-SVM) model applied to textual data. Figure 2 shows the steps of the failure analysis modeling methodology by extracting the best textual features using supervised variable selection techniques and representing the predictive models between failure description X and conclusion of failure Y for this analyzed data. This proposed methodology consists of three main phases. First, we perform the pipeline preprocessing of the failure analysis description X and conclusion of failure Y. This is a most important and time consuming part of textual data because failure to clean and prepare the data could compromise the predictive model. The Phase 2 shows the application of the Word2Vec vectorization method on preprocessed textual data to obtain numerical data.

The Phase 3 shows the application of GA variable selection method combined with decision tree or support vector machine supervised learning on vectorized preprocessed data. To quantify the accuracy of the selected predictive model on discriminate textual features, we compute the different metrics like as BLEU score and Cosine similarity. Finally we compare the predicted textual conclusion and the original textual conclusion to confirm the similarities between them.

## Application and results

All data treatments were performed using the MATLAB-R2022b environment, and scripts are available upon request.

### Data description and structure

Data description and analysis is an important phase that precedes modeling. An accurate representation of the data is necessary to define the parameters of a model. We have a textual dataset on failure analysis of Microelectronics production. The original dataset provided by STMicroelectronics dated between 2019 and 2021 consists of two parts: the first is the description of failure analysis *X* (source of failure request, properties of samples and details of failure) and the second is the dataset of its conclusion *Y* (analysis conclusion, success rate and cycle time). Tables 1 and 2 contains a list of different features of *X* and *Y* with a short description. This data has been transformed from a vertical stacking of analysis to horizontal stacking. This means that its description (objective, context, etc.) as well as its conclusion of failure represent an observation. The transformation reduces the data size to 12,300 observations and we keep 19 preprocessed features out of dates. After getting clean processed data using the preprocessing pipeline introduced in^40^, we vectorize using on Word2Vec. Genism’s Word2Vec settings are kept except the vocabulary size is set to 1000 and the minimum word is three^41^.

In formalizing our approach, we use the following notations: let $X= \{x_{ij} \}_{i=1, j=1}^{n, m}$ represents the input space of a given dataset where *n* is the number of samples and *m* is the number of features; $Y = \{y_{ij} \}_{i=1, j=1}^{n, p}$ represents the output space of conclusion failure dataset where *p* is the number of features.

**Table 1:** Textual features and its description present in the data set of the conclusion of failure analysis Y.

Feature (Y)	Description
Pt failure/Elt by sample	This is the point on the sample where an expert observes the failure during fault analysis
Macro failure mode by sample	Macro failure mode of a sample is the type of failure observed on sample before a failure analysis is requested
Elementary failure mode	Elementary failure observed during analysis
Tech cause/defect by sample	Technical cause of the failure
Analysis conclusion	Conclusion of the failure analysis

**Table 2:** Textual features and its description present in the data set of the description of failure analysis X.

Feature (X)	Description
Subject	A unique subject particular to the fault expert desire to analyses
Context	Context of the failure analysis
Objectives/work description	Objective of the fault analysis and a description of how to proceed provided by expert
Source of failure (request)	Identification of source of the failure for the component of a given sample
Source of failure (detailed)	Details of the source of the failure record for the identified failure source
Requestor	Verify a product problem and determine corrective action through the evaluation of a small quantity
Requested activity	Tries to find the wrong things in material, design, production, installation, and service
Priority level	Level of priority of the analysis
High confidentiality	Confidentiality level of failure analysis
Confidentiality	The principle of confidentiality consists of giving access to data and information only to authorized persons with a defined and specific need to see or use such information
Reference	Is the identifier that describes the location and team handling the failure product. e.g., ADG CST FA Team-19-00281 contains information about location [ADG], failure analysis team [CST FA Team], year [19] and failure analysis number [00281]
Organization	Organizational failure analysis is described for in-depth identification of organizational deficiencies and failures that can lead to accidents
Organization Division	The description of current organizations and processes that control the causes of failures
Department	The failure analysis department described the section under the FA lab where the analysis is carried out
Cost Center	The Cost Center is a department or a distinct unit or division within the framework of a company. These cost centers indirectly contribute to the organization’s profits
Site	This describes the location where the Failure Laboratory is situated e.g., Grenoble. The FA process is finished once there is enough information to make a conclusion about the location of the failure site
Lab	Determine the nature and the causes of a defect with an expertise of the product in a laboratory
Lab Team	Select the causes of a failure by the teams of people from the failure analysis laboratory
Project	The description given to the failure analysis project given by the FA team

### Preprocessing pipeline

Eliminating noise by removing whitespace and punctuation, correcting spelling errors, deleting duplicate instances, converting text to lowercase, and removing stop words and words with less than three letters are all examples of preprocessing text. We will start with the stages of the preparation pipeline:

- Symbol and alphanumeric removal: This technique removes words from the text that do not add to the intelligence pattern or the analytic sample, such as symbols and occasionally alphanumeric words. They are just stop words and inflexions that are used to emphasize meaning, thus they have been removed^42^.
- Tokenization and Thresholding: Tokenizing is to change or break the sentence into a token by using a separator^42^. Thresholding is a term used to remove words below certain length. In this paper, we set the threshold at two.
- Stemmatization and Lemmatization: this is the process of removing affixes (prefixes and suffixes) from textual features^43^.
- Abbreviation: Abbreviations are common in FRACAS, hence the need to replace them with their original meaning. We have created a dictionary of abbreviations to alleviate this challenge.

### Optimization of the parameters of the GA

A critical phase of GA is the right choice of its parameters in order to ensure the convergence of the algorithm to the optimal solution. The parameters have been initialized as follows: the number of elites $N_{e} = 2$, the fraction of crossover $F_{c} = 0.8$, the maximum number of iterations $T=100$, the population size $N = 100$ and the tolerance $\epsilon =10^{-6}$. These values have been used for several implementation of GA since they give good results for similar data^44^.

To identify the optimal values for *L* and *N*, the GA was evaluated for different sizes of chromosomes. When the algorithm has converged (tolerance $\epsilon$) or when it has reached the maximum number of iterations (T), the values chromosome size *L* that give the maximize value of the fitness function are chosen as the optimal values (Eq. 28):

28 $$\begin{aligned} L = \max _{L = 3 \cdots 8} \left\{ F(z_{i}) \right\} . \end{aligned}$$

**Figure 3:**
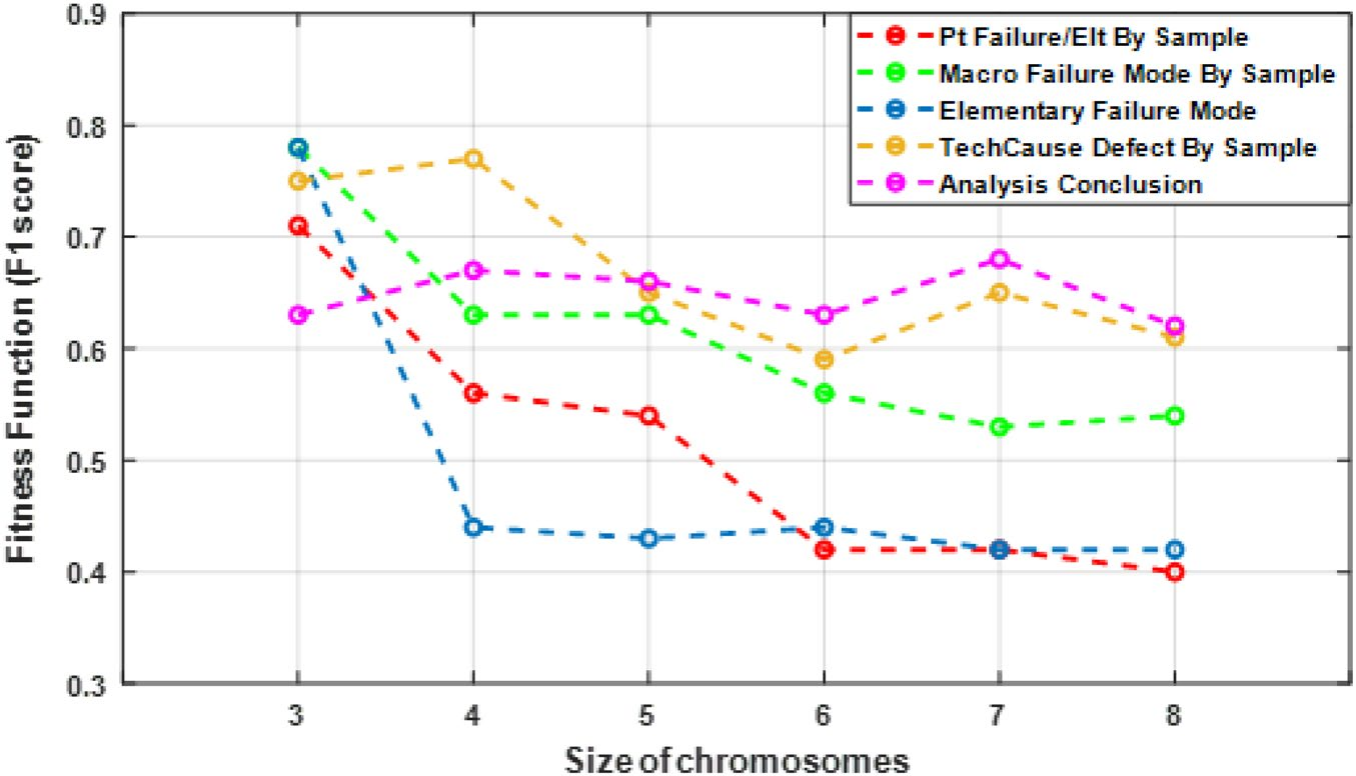
Values of GA-DT fitness functions for different sizes of L chromosomes. The optimal value is the highest F1 score.

**Figure 4:**
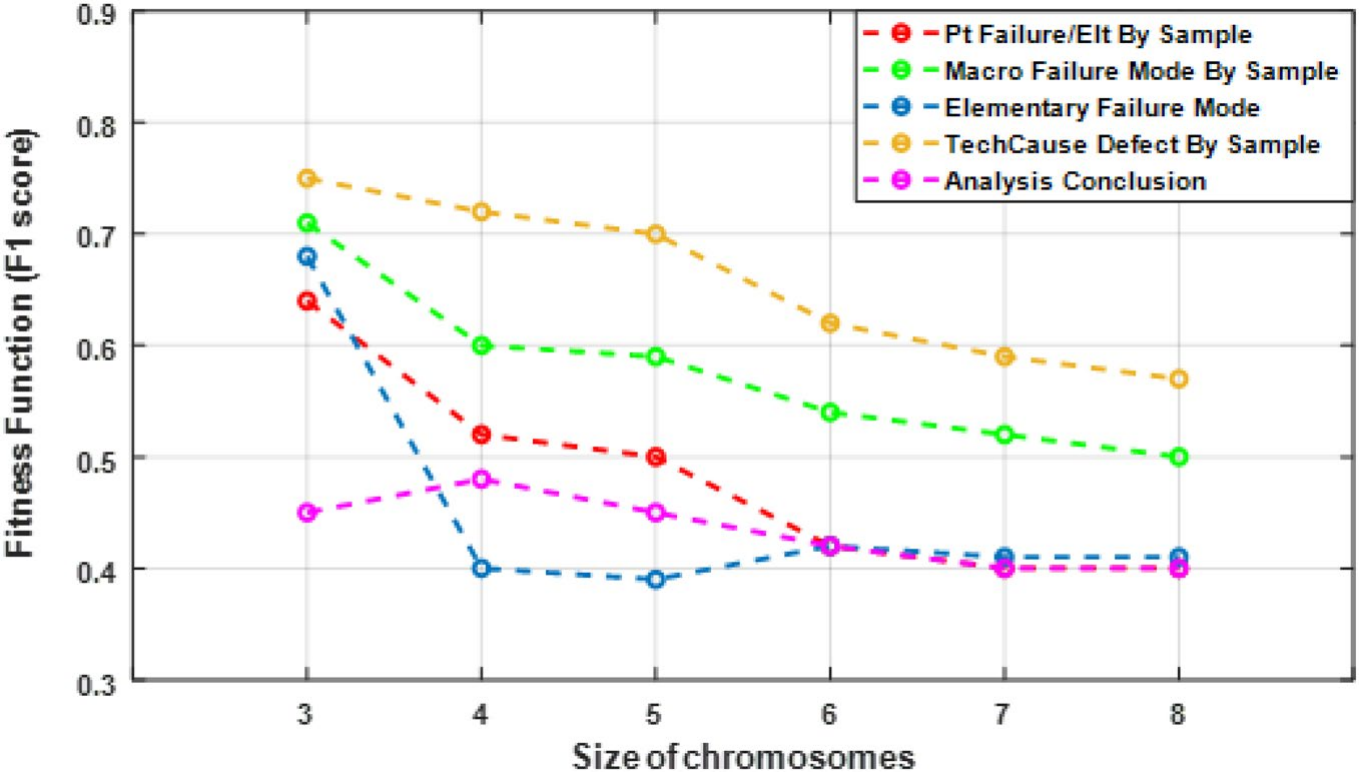
Values of GA-SVM fitness functions for different sizes of L chromosomes. The optimal value is the highest F1 score.

The best accuracy of GA-SVM and GA-DT was evaluated for different sizes of chromosomes, $L=3, \ldots , 8$. Figures 3 and 4 show the fitness values of GA-DT and GA-SVM algorithms respectively. We found that $L = 3$ or 4 gives the highest fitness value for both methods. This indicates we need all four failure description features to build the best predictive model of analysis conclusion of failure.

**Table 3:** Values of the accuracy, BLEU scores, cosine similarities for both GA-SVM and GA-DT algorithms.

Algorithm	Feature (Y)	Accuracy	BLUE score	Cosine similarity
GA-SVM	Pt failure/Elt by sample	74%	0.56	0.61
GA-DT	Pt failure/Elt by sample	**84%**	**0.72**	**0.85**
DT on all features	Pt failure/Elt by sample	70%	0.54	0.70
SVM on all features	Pt failure/Elt by sample	67%	0.48	0.52
GA-SVM	Macro failure mode by sample	75%	0.70	0.72
GA-DT	Macro failure mode by sample	**84%**	**0.95**	**0.97**
DT on all features	Macro failure mode by sample	72%	0.65	0.70
SVM on all features	Macro failure mode by sample	70%	0.62	0.65
GA-SVM	Elementary failure mode	72%	0.66	0.70
GA-DT	Elementary failure mode	**78%**	**0.95**	**0.97**
DT on all features	Elementary failure mode	69%	0.64	0.66
SVM on all features	Elementary failure mode	68%	0.62	0.64
GA-SVM	Tech cause/defect by sample	70%	0.48	0.54
GA-DT	Tech cause/defect by sample	**77%**	**0.75**	**0.93**
DT on all features	Tech cause/defect by sample	68%	0.65	0.67
SVM on all features	Tech cause/defect by sample	65%	0.60	0.62
GA-SVM	Analysis conclusion	13%	0.09	0.08
GA-DT	Analysis conclusion	**25%**	**0.32**	**0.30**
DT on all features	Analysis conclusion	8%	0.04	0.06
SVM on all features	Analysis conclusion	6%	0.02	0.05

A higher value of these metrics signifies a better predictive model within the conclusion of failure.

Significant values are given in bold.

**Table 4:** Some examples of textual samples of analysis conclusion and their three best predictions by the GA-DT algorithm.

Sample	Predict Y	BLUE score
[‘induct’, ‘reactanc’, ‘ray’, ‘nobelium’, ‘abnorm’, ‘inspect’, ‘astatin’, ‘reject’, ‘sampl’, ‘curv’, ‘trace’, ‘short’, ‘leakag’, ‘beryllium’, ‘detect’, ‘arsen’, ‘compar’, ‘sampl’, ‘scan’, ‘auger’, ‘microscopi’, ‘analysi’, ‘delamin’, ‘issu’, ‘die’, ‘attach’, ‘observ’]	(1) [‘induct’, ‘reactanc’, ‘ray’, ‘nobelium’, ‘abnorm’, ‘inspect’, ‘astatin’, ‘reject’, ‘sampl’, ‘curv’, ‘trace’, ‘short’, ‘beryllium’, ‘detect’, ‘1-trichloroethan’, ‘leakag’, ‘arsen’, ‘compar’, ‘scan’, ‘auger’, ‘microscopi’, ‘analysi’, ‘delamin’, ‘issu’, ‘die’, ‘attach’, ‘observ’, ‘intern’, ‘assembl’, ‘defect’, ‘liquid’, ‘crystal’, ‘hot’, ‘spot’, ‘nearbi’, ‘pin’, ‘failur’, ‘crater’]	0.974
	(2) [‘induct’, ‘reactanc’, ‘ray’, ‘nobelium’, ‘abnorm’, ‘inspect’, ‘astatin’, ‘reject’, ‘sampl’, ‘curv’, ‘trace’, ‘short’, ‘leakag’, ‘beryllium’, ‘detect’, ‘1-trichloroethan’, ‘arsen’, ‘compar’, ‘scan’, ‘auger’, ‘microscopi’, ‘analysi’, ‘delamin’, ‘issu’, ‘die’, ‘attach’, ‘observ’, ‘intern’, ‘assemb’, ‘defect’, ‘liquid’, ‘crystal’, ‘hot’, ‘spot’, ‘nearbi’, ‘pin’, ‘failur’, ‘local’, ‘crater’]	0.949
	(3) [‘induct’, ‘reactanc’, ‘ray’, ‘nobelium’, ‘abnorm’, ‘beryllium’, ‘observ’, ‘inspect’, ‘astatin’, ‘reject’, ‘sampl’, ‘curv’, ‘trace’, ‘short’, ‘detect’, ‘arsen’, ‘compar’, ‘scan’, ‘auger’, ‘microscopi’, ‘analysi’, ‘delamin’, ‘issu’, ‘die’, ‘attach’, ‘intern’, ‘electron’, ‘assembl’, ‘defect’, ‘liquid’, ‘crystal’, ‘hot’, ‘spot’, ‘nearbi’, ‘pin’, ‘failur’]	0.938
[‘induct’, ‘reactanc’, ‘ray’, ‘nobelium’, ‘abnorm’, ‘inspect’, ‘astatin’, ‘reject’, ‘sampl’, ‘curv’, ‘trace’, ‘short’, ‘leakag’, ‘beryllium’, ‘detect’, ‘sampl’, ‘1-trichloroethan’, ‘arsen’, ‘compar’, ‘scan’, ‘auger’, ‘microscopi’, ‘analysi’, ‘delamin’, ‘issu’, ‘die’, ‘attach’, ‘observ’, ‘intern’, ‘assembl’, ‘defect’, ‘liquid’, ‘crystal’, ‘hot’, ‘spot’, ‘nearbi’, ‘pin’, ‘failur’, ‘local’, ‘crater’]	(1) [‘induct’, ‘reactanc’, ‘ray’, ‘nobelium’, ‘abnorm’, ‘inspect’, ‘astatin’, ‘reject’, ‘sampl’, ‘curv’, ‘trace’, ‘short’, ‘leakag’, ‘beryllium’, ‘detect’, ‘1-trichloroethan’, ‘arsen’, ‘compar’, ‘scan’, ‘auger’, ‘microscopi’, ‘analysi’, ‘delamin’, ‘issu’, ‘die’, ‘attach’, ‘observ’, ‘intern’, ‘assemb’, ‘defect’, ‘liquid’, ‘crystal’, ‘hot’, ‘spot’, ‘nearbi’, ‘pin’, ‘failur’, ‘local’, ‘crater’]	0.953
	(2) [‘induct’, ‘reactanc’, ‘ray’, ‘nobelium’, ‘abnorm’, ‘inspect’, ‘astatin’, ‘reject’, ‘sampl’, ‘curv’, ‘trace’, ‘short’, ‘beryllium’, ‘detect’, ‘1-trichloroethan’, ‘leakag’, ‘arsen’, ‘compar’, ‘scan’, ‘auger’, ‘microscopi’, ‘analysi’, ‘delamin’, ‘issu’, ‘die’, ‘attach’, ‘observ’, ‘intern’, ‘assembl’, ‘defect’, ‘liquid’, ‘crystal’, ‘hot’, ‘spot’, ‘nearbi’, ‘pin’, ‘failur’, ‘crater’]	0.943
	(3) [‘induct’, ‘reactanc’, ‘ray’, ‘nobelium’, ‘abnorm’, ‘inspect’, ‘astatin’, ‘reject’, ‘sampl’, ‘1-trichloroethan’, ‘curv’, ‘trace’, ‘short’, ‘beryllium’, ‘detect’, ‘arsen’, ‘compar’, ‘ scan’, ‘auger’, ‘microscopi’, ‘analysi’, ‘delamin’, ‘issu’, ‘die’, ‘ attach’, ‘observ’, ‘intern’, ‘assembl’, ‘defect’, ‘liquid’, ‘crystal’, ‘local’, ‘hotspot’, ‘nearbi’, ‘pin’, ‘failur’, ‘crater’, ‘pend’, ‘requestor’, ‘decis’]	0.917
[‘induct’, ‘reactanc’, ‘ray’, ‘nobelium’, ‘abnorm’, ‘inspect’, ‘astatin’, ‘reject’, ‘sampl’, ‘1-trichloroethan’, ‘curv’, ‘trace’, ‘short’, ‘leakag’, ‘beryllium’, ‘detect’, ‘arsen’, ‘compar’, ‘scan’, ‘auger’, ‘microscopi’, ‘analysi’, ‘delamin’, ‘issu’, ‘die’, ‘attach’, ‘observ’, ‘intern’, ‘assembl’, ‘defect’, ‘crater’]	(1) [‘induct’, ‘reactanc’, ‘ray’, ‘nobelium’, ‘abnorm’, ‘beryllium’, ‘observ’, ‘inspect’, ‘astatin’, ‘reject’, ‘sampl’, ‘curv’, ‘trace’, ‘minor’, ‘leakag’, ‘detect’, ‘arsen’, ‘compar’, ‘transistor’, ‘outlin’, ‘packag’, ‘scan’, ‘auger’, ‘microscopi’, ‘analysi’, ‘delamin’, ‘issu’, ‘die’, ‘attach’, ‘intern’, ‘assembl’, ‘defect’, ‘liquid’, ‘crystal’, ‘hot’, ‘spot’, ‘nearbi’, ‘pin’, ‘failur’, ‘crater’]	0.912
	(2) [‘induct’, ‘reactanc’, ‘ray’, ‘nobelium’, ‘abnorm’, ‘beryllium’, ‘observ’, ‘inspect’, ‘astatin’, ‘reject’, ‘sampl’, ‘curv’, ‘trace’, ‘leakag’, ‘detect’, ‘short’, ‘1-trichloroethan’, ‘arsen’, ‘compar’, ‘scan’, ‘auger’, ‘microscopi’, ‘analysi’, ‘delamin’, ‘issu’, ‘die’, ‘attach’, ‘intern’, ‘electron’, ‘assembl’, ‘defect’, ‘liquid’, ‘crystal’, ‘hot’, ‘spot’, ‘nearbi’, ‘pin’, ‘failur’]	0.909
	(3) [‘induct’, ‘reactanc’,‘ray’, ‘nobelium’, ‘abnorm’, ‘beryllium’, ‘observ’, ‘inspect’, ‘astatin’, ‘reject’, ‘sampl’, ‘curv’, ‘trace’, ‘leakag’, ‘detect’, ‘1-trichloroethan’, ‘short’, ‘arsen’, ‘compar’, ‘scan’, ‘auger’, ‘microscopi’, ‘analysi’, ‘delamin’, ‘issu’, ‘die’, ‘attach’, ‘intern’, ‘electron’, ‘assembl’, ‘defect’, ‘liquid’, ‘crystal’, ‘hot’, ‘spot’, ‘nearbi’, ‘pin’, ‘failur’]	0.908
[‘induct’, ‘reactanc’, ‘ray’, ‘nobelium’, ‘abnorm’, ‘inspect’, ‘astatin’, ‘reject’, ‘sampl’, ‘curv’, ‘trace’, ‘short’, ‘leakag’, ‘beryllium’, ‘detect’, ‘arsen’, ‘compar’, ‘sampl’, ‘scan’, ‘auger’, ‘microscopi’, ‘analysi’, ‘delamin’, ‘issu’, ‘die’, ‘attach’, ‘observ’, ‘intern’, ‘abnorm’, ‘visual’, ‘crater’, ‘defect’]	(1) [‘induct’, ‘reactanc’, ‘ray’, ‘nobelium’, ‘abnorm’, ‘inspect’, ‘astatin’, ‘reject’, ‘sampl’, ‘curv’, ‘trace’, ‘short’, ‘beryllium’, ‘detect’, ‘1-trichloroethan’, ‘arsen’, ‘compar’, ‘transistor’, ‘outlin’, ‘packag’, ‘scan’, ‘auger’, ‘microscopi’, ‘analysi’, ‘delamin’, ‘issu’, ‘die’, ‘attach’, ‘observ’, ‘spot’, ‘intern’, ‘visual’, ‘reveal’, ‘burnt’, ‘mark’, ‘crater’, ‘defect’]	0.916
	(2) [‘induct’, ‘reactanc’, ‘ray’, ‘nobelium’, ‘abnorm’, ‘inspect’, ‘astatin’, ‘reject’, ‘sampl’, ‘curv’, ‘trace’, ‘short’, ‘leakag’, ‘beryllium’, ‘detect’, ‘1-trichloroethan’, ‘arsen’, ‘compar’, ‘scan’, ‘auger’, ‘microscopi’, ‘analysi’, ‘delamin’, ‘issu’, ‘die’, ‘attach’, ‘observ’, ‘intern’, ‘assembl’, ‘defect’, ‘liquid’, ‘crystal’, ‘decap’, ‘recov’, ‘transistor’, ‘outlin’, ‘packag’, ‘perform’, ‘crater’]	0.862
	(3) [‘induct’, ‘reactanc’, ‘ray’, ‘nobelium’, ‘abnorm’, ‘beryllium’, ‘observ’, ‘inspect’, ‘astatin’, ‘reject’, ‘sampl’, ‘curv’, ‘trace’, ‘minor’, ‘leakag’, ‘detect’, ‘arsen’, ‘compar’, ‘transistor’, ‘outlin’, ‘packag’, ‘scan’, ‘auger’, ‘microscopi’, ‘analysi’, ‘delamin’, ‘issu’, ‘die’, ‘attach’, ‘intern’, ‘assembl’, ‘defect’, ‘liquid’, ‘crystal’, ‘hot’, ‘spot’, ‘nearbi’, ‘pin’, ‘failur’, ‘crater’]	0.853
[‘induct’, ‘reactanc’, ‘ray’, ‘nobelium’, ‘abnorm’, ‘inspect’, ‘astatin’, ‘reject’, ‘sampl’, ‘curv’, ‘trace’, ‘leakag’, ‘beryllium’, ‘detect’, ‘short’, ‘sampl’, ‘arsen’, ‘compar’, ‘scan’, ‘auger’, ‘microscopi’, ‘analysi’, ‘delamin’, ‘issu’, ‘die’, ‘attach’, ‘observ’, ‘intern’, ‘assembl’, ‘defect’, ‘liquid’, ‘crystal’, ‘hot’, ‘spot’, ‘nearbi’, ‘pin’, ‘failur’, ‘local’, ‘crater’]	(1) [‘induct’, ‘reactanc’, ‘ray’, ‘nobelium’, ‘abnorm’, ‘inspect’, ‘astatin’, ‘reject’, ‘sampl’, ‘curv’, ‘trace’, ‘leakag’, ‘beryllium’, ‘detect’, ‘short’, ‘1-trichloroethan’, ‘arsen’, ‘compar’, ‘scan’, ‘auger’, ‘microscopi’, ‘analysi’, ‘delamin’, ‘die’, ‘observ’, ‘carbon’, ‘attach’, ‘transistor’, ‘consid’, ‘critic’, ‘pwsso36l’, ‘dual’, ‘chip’, ‘base’, ‘spec’, ‘intern’, ‘assembl’, ‘defect’, ‘liquid’, ‘crystal’, ‘hot’, ‘spot’, ‘nearbi’, ‘pin’, ‘failur’, ‘crater’]	0.971
	(2) [‘induct’, ‘reactanc’, ‘ray’, ‘miss’, ‘wire’, ‘inspect’, ‘astatin’, ‘reject’, ‘sampl’, ‘1-trichloroethan’, ‘nobelium’, ‘abnorm’, ‘beryllium’, ‘observ’, ‘curv’, ‘trace’, ‘short’, ‘detect’, ‘leakag’, ‘arsen’, ‘compar’, ‘scan’, ‘auger’, ‘microscopi’, ‘analysi’, ‘delamin’, ‘issu’, ‘die’, ‘attach’, ‘intern’, ‘assembl’, ‘defect’, ‘electron’, ‘reveal’, ‘liquid’, ‘crystal’, ‘hot’, ‘spot’, ‘nearbi’, ‘pin’, ‘failur’, ‘crater’]	0.862
	(3) [‘induct’, ‘reactanc’, ‘ray’, ‘nobelium’, ‘abnorm’, ‘inspect’, ‘astatin’, ‘reject’, ‘sampl’, ‘curv’, ‘trace’, ‘short’, ‘leakag’, ‘beryllium’, ‘detect’, ‘1-trichloroethan’, ‘arsen’, ‘compar’, ‘scan’, ‘auger’, ‘microscopi’, ‘analysi’, ‘delamin’, ‘issu’, ‘die’, ‘attach’, ‘observ’, ‘intern’, ‘assemb’, ‘defect’, ‘liquid’, ‘crystal’, ‘hot’, ‘spot’, ‘nearbi’, ‘pin’, ‘failur’, ‘local’, ‘crater’]	0.823

### Results and discussion of the proposed methodology for the prediction of the conclusion of failure

The proposed methodology has been applied with two different fitness functions (SVM and DT). After selecting variables by the GA-SVM and GA-DT algorithms, we calculated the accuracy (%) to evaluate the performance of a predictive model, BLEU score and cosine similarity as metrics in order to quantify the results of the prediction of the conclusion of failure.

Accuracy is the most intuitive performance measure and it is simply a ratio of correctly predicted document to the total documents.

29 $$\begin{aligned} \text {Accuracy}= \frac{TP + TN}{TP + FP + FN + TN}. \end{aligned}$$

Where TP is True Positives, TN is True Negatives, FP is False Positives and FN is False Negatives. FP and FN, these values occur when the actual documents contradicts with the predicted documents. These values (BLEU score, Cosine similarity and Accuracy), presented in Table 3, confirm that the GA-DT allows a better predictive model of the textual samples to predict the failure conclusion (features Y) compared to the other algorithm such as GA-SVM. We can see that the first four features of Y give good precision and good values of BLEU score and cosine similarity for GA-DT method except the last textual feature which is the conclusion of the analysis because each sample recorded on this variable is a large textual paragraph. About this latter feature we can say that the metrics calculated (accuracy = 25%; BLEU = 0.32; Cosine = 0.30) are very good compared to the other studies on textual dataset. One can also find that the application of variable selection by the genetic algorithm improves the accuracy of the model. These results are showed in Table 3.

In Table 4 we present some examples of results obtained after the application of the genetic algorithm with decision tree (GA-DT). We display the three best predictions for each failure analysis conclusion text sample. Then, we calculate the BLEU score to quantify the similarity between these predicted samples and the original sample. One can find that the values of BLEU scores are very close to one. This indicates strong similarity between the predicted samples and the reference ones.

## Conclusion

We have proposed a methodology based on the association of a genetic algorithm with some supervised classifier methods for identification of discriminant textual features for the study of the best predictive model of failure conclusion using the features of failure descriptions.

The implementation of a genetic algorithm with a decision tree classifier as the fitness function led to the identification of a few interesting features. The BLUE score and the cosine similarity are used to evaluate the similarity between a predictive documents and a set of reference documents. We obtained very interesting values that indicate a strong similarity between the predictive documents and the references. We have also found that the application of variable selection by the genetic algorithm improve the accuracy and the metrics of the model obtained by DT or SVM methods.

We have shown that the discriminating features selected by the proposed GA-DT method provide the best predictive model of the failure conclusion according to the description of the failure process compared to GA-SVM model or the direct application of the decision tree or the support vector machine applied to all the features of the description of the failure (i.e., without any preselection method). As a perspective, we are working towards addressing the following challenges: 1) Improving the performance of the model by applying a generative sequence-to-sequence language model for failure conclusion generation given failure description; 2) Propose a methodology based on Genetic algorithm (GA) with decision tree (DT) to select the most important input variables that best predicts the conclusion (root cause) of a Failure analysis (FA). These variables will then be used to train a Transformer model for failure conclusion generation such as GPT2 transformer model etc.

## Data Availability

All data, models, and code generated or used during the study appear in the submitted article and are provided upon request by contacting Abbas Rammal via email: rammal_abbass@hotmail.com.
